# Impact of kidney function on stiffness of small conduit arteries in hypertension and obesity

**DOI:** 10.1097/HJH.0000000000003957

**Published:** 2025-02-07

**Authors:** Diego Moriconi, Monica Nannipieri, Smriti Badhwar, Stefano Taddei, Pierre Boutouyrie, Rosa Maria Bruno

**Affiliations:** a Department of Clinical and Experimental Medicine, University of Pisa, Pisa, Italy; b PARCC INSERM; c Service de Pharmacologie, AP-HP, Hôpital Europeen Georges Pompidou; d Université Paris Cité, Inserm, PARCC, F-75015 Paris, France

**Keywords:** arterial stiffness, estimated glomerular filtration rate, obesity, small arteries conduit

## Abstract

**Background:**

Arterial stiffness is a key cardiovascular risk factor influenced by conditions like hypertension, obesity and kidney function. Although large arteries have been extensively studied, small conduit arteries remain less investigated. This study aims to explore the impact of kidney function on small conduit artery stiffness in two distinct groups: normotensive individuals with severe obesity and normal-weight hypertensive individuals.

**Methods:**

Thirty-three severely obese (OB) individuals, 33 hypertensive (HT) individuals, and 33 normotensive, normal-weight control participants, matched for age and sex, were recruited. Eleven participants (33%) in both the OB and HT groups had estimated glomerular filtration rate (eGFR) less than 60 ml/min/1.73 m^2^. Aortic stiffness (carotid–femoral pulse wave velocity) was recorded. Ultrasound images of common carotid, radial, and interdigital arteries were acquired for the assessment of geometry, distensibility coefficient, circumferential wall stress, and Young's elastic modulus (Einc).

**Results:**

The OB group exhibited higher radial stiffness (both Einc and distensibility coefficient) compared to the HT and control groups, independent of radial diameter adjustments. An inverse correlation between eGFR and radial Einc was noted only in the OB group (*P* = 0.002). Conversely, a direct correlation between eGFR and carotid distensibility coefficient was found only in the HT group (*P* = 0.001). In multivariable analysis, eGFR and BMI were the only predictors of radial Einc in the overall population.

**Conclusion:**

Severe obesity and reduced eGFR synergistically increase radial artery stiffness, a phenomenon not observed in essential hypertension. This study suggests that moderate chronic kidney disease exacerbates vascular alterations in obese individuals, highlighting the need for further research on the role of small conduit arteries in cardiovascular risk.

## INTRODUCTION

Arterial stiffness is an important cardiovascular risk marker, and its relationship with various factors such as hypertension, obesity, and renal function is of significant clinical interest [1].

Although arterial stiffness has been predominantly studied in large arteries, particularly the aorta, small conduit arteries have been less extensively investigated until now.

In middle-sized/small conduit arteries unaffected by atherosclerosis, like the radial arteries, essential hypertension does not appear to alter elasticity. Notably, a study found that hypertensive individuals exhibited even lower stiffness compared to normotensive counterparts under isobaric conditions [2]. Recently, our research group identified increased stiffness in small conduit arteries among individuals with severe obesity and normal kidney function, as compared to a matched normal-weight group. This phenomenon was observed as a reduction in distensibility in both radial and digital arteries, even after adjusting for age and mean blood pressure (MBP) [3]. We hypothesize that oxidative stress and low-grade inflammation, conditions commonly associated with morbid obesity [4], may contribute to excessive arterial fibrosis and extracellular matrix deposition, thus accelerating stiffness in small conduit arteries. Additionally, individuals with end-stage renal disease (ESRD) show significantly increased stiffness in small conduit arteries compared to both hypertensive and normotensive individuals with normal renal function. This increase in stiffness is observed regardless of whether it is expressed at similar wall stress or at a given blood pressure [2]. The structural changes seen in ESRD, including vascular calcifications, are distinct from those observed in aging or atherosclerosis and are consistently associated with ‘uremia’ rather than high blood pressure [5].

To date, no studies have investigated the impact of renal function on small conduit arteries in the context of severe obesity and essential hypertension. Based on this background, the aim of this study is to investigate the impact of mild to moderate chronic kidney disease (CKD) on small conduit artery stiffness in two distinct subgroups: individuals with severe obesity without essential hypertension and normal-weight hypertensive individuals.

## MATERIALS AND METHODS

Thirty-three individuals affected by morbid obesity (BMI ≥ 35 kg/m^2^) on the waiting list for bariatric surgery were recruited from the Outpatients’ Clinic for metabolic diseases of the University Hospital of Pisa (obese (OB) group). These patients performed vascular tests as part of their routine work-up for cardiovascular risk stratification before bariatric surgery.

Additionally, 33 participants with normal weight (18 < BMI ≤ 25 kg/m^2^) and essential hypertension (hypertensive (HT) group) and 33 participants normotensive with normal weight (CONTROL group), matched by age and sex, were enrolled in the same hospital as part of the ‘Very High-Frequency Ultrasonography for arterial phenotyping in patients with Cervico-Cerebral Artery Dissection (CCeAD), Hypertension, Spontaneous Coronary Artery Dissection (SCAD) and FibroMuscular DysplasIA (FMD)’ – FUCHSIA study.

The exclusion criteria for all groups included the presence of type 2 diabetes, endocrinopathies, cancer, secondary causes of hypertension, heart or liver disease.

All participants underwent a detailed medical history review and a comprehensive physical examination.

The study was conducted in accordance with the guidelines of the Declaration of Helsinki and was approved by the Institutional Ethics Committee. Informed written consent was obtained from all participants prior to enrolment.

### Experimental session

All participants underwent a detailed medical history review and a comprehensive physical examination. Venous blood samples were collected after an overnight fast for standard biochemical analyses.

Office blood pressure was measured at the brachial level with patients resting in the supine position for at least 10 min, using an automatic oscillometric device (OMRON-705IT, Omron Corporation, Kyoto, Japan) with an appropriate adult-size cuff according to arm circumference. The device was validated according to the Association for the Advancement of Medical Instrumentation/European Society of Hypertension/International Organization for Standardization (AAMI/ESH/ISO) protocol. Blood pressure measurements were repeated at least three times at 2-min intervals. Hypertension was diagnosed using the European guidelines [6].

Carotid ultrasound scans were acquired using high-resolution B-mode ultrasound with a at least 7.5 MHz linear array transducer by a trained operator (MyLab25, Esaote, Florence, Italy). The systolic and diastolic diameters of the left and right carotid arteries were measured on the distal wall 1–2 cm proximal to the bifurcation. Radial and interdigital diameter and thickness were assessed using ultrahigh-frequency ultrasound with a Vevo MD (FUJIFILM VisualSonics Inc., Toronto, Ontario, Canada) equipped with a 70 MHz probe (spatial resolution of 30 μm).

Edge detection and contour tracking software (Cardiovascular Suite, Quipu srl) was used for both high-resolution ultrasound images of the carotid arteries and ultrahigh-frequency ultrasound of the radial and interdigital arteries to obtain geometry and distensibility for each site. This automated system has been developed and validated for carotid measurements with standard ultrasound and has recently been shown to be accurate with ultrahigh-frequency ultrasound clips.

The distensibility coefficient (DC) was calculated using the formula DC = Δ*A*/Ad ∗ Δ*P*, where Ad is the diastolic lumen area, Δ*A* is the stroke change in lumen area (As − Ad, with As being the systolic lumen area), and Δ*P* is aortic pulse pressure (aPP) for the carotid artery, and brachial pulse pressure (PP) for the radial and interdigital arteries. Compliance (CC) was measured as CC = 2 ∗ (Ds − Dd)/Ds/Δ*P*, where Dd and Ds are the diastolic and systolic diameters, respectively.

Arterial remodeling was estimated using the wall-to-lumen ratio (WLR) and the wall cross-sectional area (WCSA). WLR was calculated as 2 ∗ IMT/(Dd − 2 ∗ IMT), where IMT is the intima–media thickness. WCSA was estimated as Ad − LCSA, where LCSA is *π* ∗ (Dd − 2 ∗ IMT)^2^/4, representing the vascular lumen area.

Mean circumferential wall stress (*ζθ*, in kPa) was calculated according to Lamé's equation as follows: *ζθ* = MBP × (1/WLR), where MBP is mean blood pressure, calculated as DBP + 0.4 ∗ pulse pressure. Specifically, aPP was used for the carotid artery, whereas PP was applied for the radial and interdigital arteries.

The incremental elastic modulus (Einc), which provides direct information about the elastic properties of the wall material independent of vessel geometry, was calculated using the formula Einc = [3 ∗ (1 + LCSA/WCSA)]/DC, as previously described [7].

Peripheral pressure waveforms were recorded from the carotid and radial arteries at the wrist using applanation tonometry. A validated generalized transfer function (SphygmoCor software) was used to generate corresponding central aortic pressures and pressure waveforms. Waveforms were calibrated with brachial MBP, calculated as DBP + 0.4 ∗ PP.

Augmentation pressure (augmented pressure), the difference between the second and first systolic peak of waveforms was determined, and the Augmentation Index (AIx) was calculated as the ratio of augmented pressure to pulse pressure (PP), expressed as a percentage.

An in-device quality rating of at least 80% was required for all recordings.

Aortic stiffness was assessed by carotid–femoral pulse wave velocity (cf-PWV) as previously described. Waveforms were recorded at the femoral and carotid sites using SphygmoCor (AtCor Medical, Sydney, New South Wales, Australia). A probe or cuff was placed on the selected artery while 10–15 subsequent heartbeats were recorded. PWV was calculated from the pulse transit time (TT) using 80% of the direct distance method: PWV = 0.8 ∗ d/TT, where *d* is the surface distance between the carotid and femoral sites of measurement. The direct distance was determined using a rigid sliding caliper as previously described [8].

Kidney function assessment: serum concentration of creatinine was measured with Jaffé method traceable to IDMS reference method (CREA Roche/Hitachi 917, Mannheim, Germany). The eGFR was calculated using the Chronic Kidney Disease Epidemiology Collaboration formula (CKD-EPI 2021).

### Statistical analysis

The results were expressed as mean ± SD for normally distributed variables and as median [interquartile range] for variables with skewed distribution. Categorical variables were analyzed with the *χ*^2^ test. Continuous variables with a normal distribution were compared between the three groups (OB, HT, CONTROL) by the Student *t* test, while the skewed variables by Kruskal–Wallis test.

When significant differences in stiffness parameter were found at unadjusted analysis, variables were further compared by ANCOVA adding the arterial diameter as covariate, in order to exclude the possibility that variations in elasticity were merely due to vessel size, in accordance with the laws of arterial mechanics [9].

In univariate analysis, Pearson's test was used to explore correlations among continuous variables.

Finally, two multiple regression models, including respectively, radial Einc and carotid-DC as dependent variable, eGFR and BMI as independent variable and adjusting for age, MPB and sex were built. The Variance Inflation Factor (VIF) was used as measure of collinearity among predictor variables. Only variables with a VIF less than 3 were included in the model.

Statistical tests were performed using JMP Pro 17.3.0 (SAS Institute Inc., Cary, North Carolina, USA) using a two-sided *α* level of 0.05.

## RESULTS

### Clinical features of participants enrolled

The three groups (OB, HT and CONTROL) were matched for age and sex, with a predominance of women (as more women undergo bariatric surgery). There were no differences in smoking habits between groups, while the prevalence of dyslipidemia was similar between the OB and HT groups (Table 1). Eleven participants (33%) in both the OB and HT groups had eGFR less than 60 ml/min/1.73 m^2^, indicating they were affected by CKD-G3.

**TABLE 1:** Anthropometrics and clinical features of participants

	OB (*n* = 33)	HT (*n* = 33)	CONTROL (*n* = 33)	*P* value
Age (years)	52 ± 10	53 ± 13	51 ± 13	*0.634*
Sex (m/f)	9/24	9/24	10/23	*0.951*
Smoking (*n*,%)	4 (12)	4 (12)	3 (9)	*0.975*
Dyslipidemia (*n*,%)	10 (30)	9 (27)	0 (0)	*0.003*
CKD (*n*,%)	11 (33)	11 (33)	0 (0)	*0.001*
BMI (kg/m^2^)	42.3 ± 6.8	23.9 ± 3.0	22.5 ± 2.6	*<0.001*
SBP (mmHg)	124 ± 10^a^	134 ± 13^a^^,^^b^	114 ± 12	*<0.001*
DBP (mmHg)	76 ± 8^a^	77 ± 9^a^	68 ± 8	*<0.001*
MBP (mmHg)	92 ± 7^a^	96 ± 9^a^	83 ± 9	*<0.001*
PP (mmHg)	48 ± 10^a^	56 ± 10^a^^,^^b^	46 ± 8	*<0.001*
HR (bpm)	72 ± 11^a^	69 ± 10	64 ± 8	*<0.001*
aSBP (mmHg)	114 ± 10^a^	123 ± 13^a^^,^^b^	103 ± 12	*<0.001*
aDBP (mmHg)	78 ± 6^a^	78 ± 8^a^	67 ± 9	*<0.001*
aMBP (mmHg)	90 ± 6^a^	93 ± 9^a^	78 ± 10	*<0.001*
aPP (mmHg)	37 ± 8^a^	45 ± 9^a^^,^^b^	36 ± 8	*<0.001*
AIx@75 (%)	24.8 ± 14.1	25.6 ± 12.4	19.8 ± 14.5	*0.198*
AP (%)	9.6 ± 6.8	13.3 ± 6.6^a^^,^^b^	9.8 ± 6.6	*0.012*
cf-PWV (m/s)	8.4 ± 1.4^a^	8.0 ± 1.8^a^	7.1 ± 1.6	*0.002*
eGFR (ml/min/1.73m^2^)	84 ± 30^a^	80 ± 28^a^	96 ± 14	*0.037*

aDBP, aortic DBP; Aix@75%, augmentation index corrected for heart rate; AP, augmented pressure; aSBP, aortic blood pressure; cf-PWV, carotid–femoral pulse wave velocity; HT, hypertensive; MBP, mean blood pressure; OB, obese; PP, pulse pressure.

a <0.05 vs. CONTROL.

b <0.05 HT vs. OB.

Concerning brachial and aortic blood pressure parameters, we observed a progressive increase of systolic component from the CONTROL group to the HT group. Both brachial and aortic DBP was similar in the OB and HT groups and higher compared to CONTROL, as well as aortic and brachial MBP. Meanwhile, pulse pressure (PP, aPP) was higher in the HT group compared to the other two groups.

There were no differences in AIx between the groups, while cf-PWV was similarly increased in OB and HT groups compared to CONTROL group (Table 1).

### Arteries’ structure and functional parameters

The main arterial parameters of carotid, radial and interdigital arteries are reported in Table 2.

**TABLE 2:** Arterial parameters of carotids, radials and interdigital in obese, hypertensive and control groups

	OB (*n* = 33)	HT (*n* = 33)	CONTROL (*n* = 33)	*P* value
Carotid				
D-diameter (mm)	7.19 ± 0.78^a^	7.21 ± 1.02^a^	6.69 ± 0.68	*0.032*
IMT (mm)	0.62 ± 0.21	0.66 ± 0.17	0.60 ± 0.11	*0.327*
DC (10^−3^ / kPa)	23.8 ± 14.0^a^	21.6 ± 9.0^a^	29.4 ± 12.4	*0.010*
CC (m^2^ × kPa^−1^ × 10^−6^)	0.84 ± 0.33	0.80 ± 0.29	0.92 ± 0.33	*0.267*
WLR	0.23 ± 0.09	0.24 ± 0.05	0.23 ± 0.05	*0.886*
WCSA (m^2^/10^2^)	12.0 ± 4.3	13.6 ± 4.8^a^	11.2 ± 3.0	*0.043*
Einc (kPa 10^3^)	0.52 ± 0.32^b^	0.47 ± 0.19	0.36 ± 0.13	*0.029*
*ζθ* (kPa)	62.6 ± 25.3	55.7 ± 13.8	47.5 ± 9.3	*0.089*
Radial				
D-diameter (mm)	2.51 ± 0.54^b^^,^^a^	2.11 ± 0.36	2.10 ± 0.32	*<0.001*
IMT (mm)	0.20 ± 0.05^a^	0.20 ± 0.05^a^	0.16 ± 0.04	*0.001*
DC (10^−4^/kPa)	19.0 ± 9.2^b^^,^^a^	30.0 ± 18.5	34.2 ± 16.0	*0.001*
CC (m^2^ × kPa^−1^ × 10^−7^)	0.09 ± 0.05	0.09 ± 0.05	0.11 ± 0.05	*0.211*
WLR	0.20 ± 0.05	0.27 ± 0.08^a^^,^^b^	0.20 ± 0.07	*0.001*
WCSA (mm^2^)	1.45 ± 0.60^a^^,^^b^	1.19 ± 0.43^a^	0.95 ± 0.27	*<0.001*
Einc (kPa 10^3^)	0.67 ± 0.36^a^^,^^b^	0.40 ± 0.27	0.37 ± 0.21	*<0.001*
*ζθ* (kPa)	66.5 ± 18.4^b^	54.5 ± 17.1	62.4 ± 15.2	*0.025*
Digital				
D-diameter (mm)	1.12 ± 0.19^b^	0.96 ± 0.20	0.98 ± 0.23	*0.012*
IMT (mm)	0.15 ± 0.04	0.16 ± 0.06	0.13 ± 0.03	*0.380*
DC (10^−4^/kPA)	19.3 ± 13.5^b^	38.2 ± 33.5	34.1 ± 24.1	*0.021*
CC (nm^2^ × kPa^−1^)	17.0 ± 11.0	19.6 ± 11.1	21.8 ± 14.2	*0.370*
WLR	0.37 (0.27–0.51)	0.41 (0.35–0.69)	0.40 (0.27–0.68)	*0.336*
WCSA (mm^2^)	0.43 ± 0.10^a^	0.36 ± 0.17	0.33 ± 0.11	*0.042*
Einc (kPa 10^3^)	0.35 (0.24–0.81)	0.20 (0.10–0.63)	0.27 (0.15–0.47)	*0.142*
*ζθ* (kPa)	34.4 ± 14.6	28.4 ± 14.5	32.5 ± 18.0	*0.437*

ζθ, circumferential wall stress; CC, compliance; DC, distensibility coefficient; D-diameter, diastolic diameter; Einc, Young's modulus; IMT, intima–media thickness; WCSA, wall cross-sectional area; WLR, wall-to-lumen ratio.

a <0.05 vs. control.

b <0.05 HT vs. OB.

Concerning the carotid arteries, the diameter was increased in both HT and OB groups compared to CONTROL, while the WCSA was increased only in HT group. No differences were found in WLR between the three groups. Carotid DC was reduced, and the Einc was increased in both OB and HT groups compared to CONTROL group. Interestingly, Einc remained higher in OB and HT group compared to CONTROL even after adjustment of the mean carotid diameter (*P* = 0.028), while the difference in the carotid DC between all the three groups was no longer observed (*P* = 0.211). The circumferential wall stress was quite similar in all the three groups.

Concerning medium-sized arteries, in OB group, radial artery diameter was larger compared to the others, as well as the WCSA, while IMT was similarly increased in both the HT and OB groups compared to the CONTROL group. Radial DC was lower, and Einc was higher in OB group than both HT and CONTROL groups. After adjustment for the mean radial diameter, Einc remain higher in OB group compared to HT and CONTROL (*P* = 0.011), while the difference in the radial DC between the OB and HT groups was no longer observed (*P* = 0.099), but it persisted between the OB and CONTROL groups (*P* = 0.001). Finally, the OB group also had a higher CWS compared to the other groups (Table 2), but this difference was attenuated after adjusting for the mean radial diameter (*P* = 0.065).

Similarly to what was observed in the radial arteries, the diastolic diameter and WCSA of the interdigital arteries were higher in the OB group than in the other two groups, while IMT and WLR were similar across all groups. Interdigital DC was significantly lower in the OB group compared to the other groups, but this difference was no longer observed after adjusting for the mean interdigital diameter (*P* = 0.211). No differences were observed in Einc and in circumferential wall stress (Table 2).

### Association between renal function and vascular parameters in obese and hypertensive individuals

**FIGURE 1:**
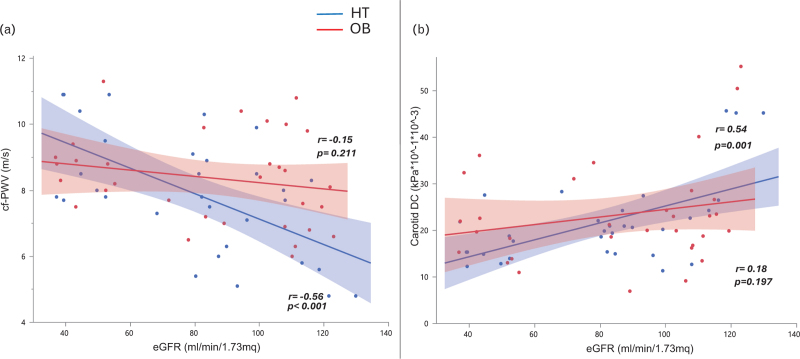
Linear correlation between estimated glomerular filtration rate and carotid–femoral pulse wave velocity (a) and between estimated glomerular filtration rate and carotid-distensibilty coefficient (b). The red points and line represent the OB group, while the blue points and line represent the HT group. HT, hypertensive; OB, obese.

As expected, there was a significant univariate correlation between eGFR and large artery stiffness evaluated with both cf-PWV and carotid-DC only in the HT group. In contrast, in the eGFR-matched OB group, no significant associations were found between eGFR and both cf-PWV and carotid-DC (Fig. 1). Furthermore, a significant association was found between eGFR and WCSA in HT groups (*P* = 0.002), while in OB group. this association showed a trend (*P* = 0.069).

**FIGURE 2:**
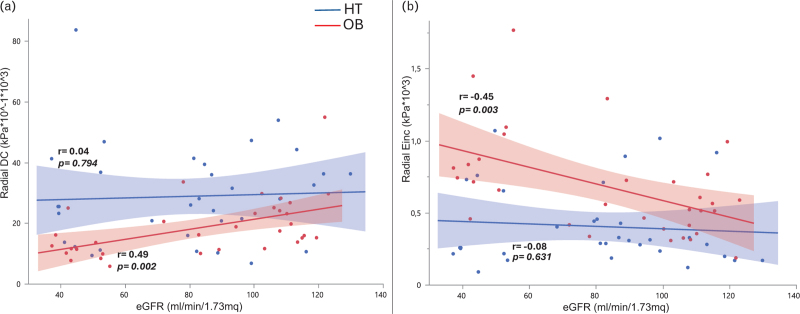
Linear correlation between estimated glomerular filtration rate and radial-distensibilty coefficient (a) and between estimated glomerular filtration rate and radial Einc (b). The red points and line represent the OB group, while the blue points and line represent the HT group. HT, hypertensive; OB, obese.

The opposite was true for the radial artery: there was a significant direct correlation between eGFR and radial-DC, and an inverse correlation between eGFR and Einc, only in the OB group but not in the HT group (Fig. 2). No significant association were found between eGFR and radial WCSA in both OB and HT groups.

Concerning the digital arteries, no correlations were found between eGFR and stiffness parameters (Einc and digital-DC), both in OB and HT groups.

Multiple regression analysis in overall population showed that eGFR (*P* = 0.033) and BMI (*P* < 0.001) were the only independent determinants of radial Einc, in a model including age, MPB and sex as confounders (Table 3a). Furthermore, also the interaction factor eGFR ∗ BMI was significant (*P* = 0.048).

**TABLE 3:** Multiple regression models considering (a) radial artery Young's modulus and (b) carotid distensibility coefficient as dependent variables, BMI and estimated glomerular filtration rate as independent variable, adjusting for age, sex and mean blood pressure

(a)				
	Coefficient	Std. error	*t*-Stat	*P* value
Age	0.001	0.003	0.48	0.632
Sex (F)	0.026	0.032	0.79	0.429
BMI	0.012	0.003	4.22	<0.001^∗^
eGFR	−0.003	0.001	−2.16	0.033^∗^
MBP	−0.003	0.003	−1.13	0.260
eGFR ∗ BMI	<−0.001	<0.001	−2.00	0.048^∗^

(b)				
	Coefficient	Std. error	*t*-Stat	*P* value
Age	−0.289	0.102	−2.89	0.006^∗^
Sex (F)	0.585	1.116	0.52	0.601
BMI	−0.120	0.100	−1.20	0.235
eGFR	0.050	0.047	1.09	0.280
aMBP	−0.277	0.100	−2.78	0.007^∗^

aMBP, aortic mean blood pressure; DC, distensibility coefficient; Einc, Young's modulus; MBP, brachial mean blood pressure.

∗ <0.05

On the contrary, the only independent determinants of carotid-DC in the same multivariable model resulted in MBP and age (Table 3b).

## DISCUSSION

The main findings of this study indicate that vascular aging induced by moderate chronic kidney disease (CKD) appears to differ in severe obesity compared to essential hypertension.

Specifically, there is an inverse association between kidney function and the stiffness of radial arteries in severe obesity. Furthermore, both higher BMI and reduced eGFR appear to synergistically contribute to radial stiffness. Conversely, in normal weight hypertension, there is an inverse association between eGFR and the stiffness of large musculo-elastic arteries, such as carotid arteries, but no association between kidney function and small conduit arteries (radial or interdigital arteries) was found.

It is known that obesity is associated with changes in the composition of the arterial wall, including increased collagen deposition and accumulation of extracellular matrix [10]. Additionally, insulin resistance can promote the growth of vascular smooth muscle cells and collagen production [11], and all these factors can collectively contribute to arterial stiffness.

Our group recently demonstrated increased radial stiffness in a cohort with severe obesity and normal kidney function compared to a normal-weight cohort [3].

In the present study, we confirmed higher radial Einc in individuals with morbid obesity and a broader range of kidney function (OB group) compared to sex-matched and age-matched hypertensive (HT) and CONTROL (C) groups, even after adjusting for mean radial diameter. This indicates that obesity causes intrinsic changes in the elastic properties of the arterial wall beyond structural alterations.

However, for the first time, we also found a significant direct association between the reduction of estimated glomerular filtration rate (eGFR) and radial stiffness within the OB group.

Obesity and CKD share common physiopathological denominators, such as chronic inflammation and oxidative stress [12–14], which could exacerbate alterations in arterial structure and impair vascular tissue regeneration. The synergistic impact of obesity and CKD on small conduit arteries has been shown by ultra-high frequency ultrasound (UHFUS) in children with ESRD, though limited to wall thickening [15]. Our study extended those findings to arterial mechanics.

The fact that ESRD is associated with increased radial artery stiffness has been known for a long time [2]. In the present study, we extend these results by showing that even moderate CKD is associated with increased radial stiffness in obesity, while it does not appear to play a predominant role in radial stiffness in essential hypertension.

One possible explanation is that, in essential hypertension, any potential increase in radial artery stiffness induced by CKD is counterbalanced by a paradoxical, blood pressure-related, increase in elasticity. Indeed, Laurent *et al.* demonstrated that not only radial artery compliance was not reduced in hypertensive patients compared to that observed at the same level of blood pressure or circumferential stress in age-matched and sex-matched normotensive individuals but was even found to be increased under isobaric conditions [16]. Being the muscular component more prominent in small conduit arteries than large arteries, the hypertrophy of vascular smooth muscle associated with hypertension may decrease the proportion of less extensible connective tissue in the vessel wall and thereby promoting increased compliance [16,17]. In contrast, in large elastic arteries, such as the carotid artery, age and hypertension rather induce fragmentation of elastic fibers and collagen deposition, thus increasing their stiffness [17].

Therefore, while in hypertension, the elasticity of the radial artery tends to increase, this phenomenon is balanced in the presence of moderate CKD, where stiffness tends to increase. Only once ESRD is reached, stiffness is likely to be increased regardless of blood pressure control, as demonstrated by Mourad *et al*. [2]. In fact, chronic kidney disease-mineral and bone disorder, characterized by disturbances in calcium and phosphate levels with increase in PTH, can contribute not only to aortic stiffness but also to radial stiffness, similarly to what was observed in primary hyperparathyroidism [18]. These effects begin when eGFR falls below 60 ml/min/1.73 m^2^ but become clinically significant when it drops around 30 ml/min/1.73 m^2^[19]. Furthermore, persistent low-grade inflammation, considered a hallmark feature of CKD and associated with the development of vascular fibrosis and stiffness, starting at eGFR less than 60 ml/min/1.73 m^2^, but it increases exponentially only in ESRD [20,21].

All these mechanisms could explain, in our opinion, why the impact of CKD on radial stiffness is maximal and independent of blood pressure values only in ESRD.

In this study, we also observed that obesity-induced changes in the arterial geometry, namely hypertrophic outward remodeling, are present not only in large arteries [22] but also in the small conduit arteries. Individuals affected by severe obesity tend to have increased total blood volume and a greater demand for blood flow, causing compensatory adaptation of the arteries to maintain adequate tissue perfusion [23]. Flow-induced remodeling (inward vs. outward) depends on vasoregulation and smooth muscle cell activity, which regulate blood vessel diameter and respond to mechanical cues essential for extracellular matrix remodeling [24]. This may lead to arterial outward remodeling to accommodate the increased blood volume and reduce vascular resistance, as wall shear stress decreases with luminal enlargement [25]. Conversely, in hypertensive participants, the increased blood pressure leads to thickening of the arterial wall and inward remodeling to resist high pressures [26], as our study confirms too.

Until now, medium-sized arteries have not been extensively explored noninvasively, largely because of the limited spatial resolution of standard ultrasound machines. Consequently, the clinical implications of increased stiffness in radial arteries, and more broadly in small conduit arteries, are not fully understood but may offer valuable insights into vascular adaptations, remodeling, and modifications in wall ultrastructure associated with cardiovascular risk factors. Unlike large arteries, they share histological and functional similarities with coronary arteries, which are pivotal for understanding cardiovascular diseases. For example, in a longitudinal study performed in 416 patients with suspected CAD, referred to myocardial perfusion scintigraphy and followed up for about 3 years, radial IMT outperformed carotid IMT and was an independent predictor of cardiovascular events on top of classical risk factors [27].

Radial artery investigation may have an additional advantage compared to carotid artery, being easy to investigate even in the presence of higher BMI and larger neck circumference. Indeed, unlike previous studies [13,28], we were not able to show the expected significant increase in carotid stiffness with declining eGFR because of greater measurement dispersion compared to HT and C groups, despite a similar correlation coefficient. For this reason, in obese patients, the measurement of the radial artery may gain greater importance in the future, as a measure of arterial stiffness and its prognostic role will be crucial to investigate in longitudinal studies.

This study has several limitations to address. First, the cross-sectional design precludes making causal inferences. Additionally, the study population was predominantly woman, reflecting the higher prevalence of women undergoing bariatric surgery, which might limit the generalizability of the findings to men.

As perspectives, we would emphasize two key points. First, our findings suggest that assessing small conduit artery stiffness may be useful to improve cardiovascular risk stratification in populations with severe obesity and CKD. For this reason, future validations through large longitudinal studies are necessary before they enter routine clinical work-up. Second, although small conduit arteries share histological similarities with coronary arteries and their thickness may predict future coronary events [27], to date, no studies have investigated the relationship between small conduit artery stiffness and coronary artery disease (CAD), highlighting the need for future research to determine its potential as a reliable marker for CAD in individuals with obesity and CKD.

In conclusion, this study highlights for the first time the differential impacts of severe obesity and hypertension on radial artery stiffness, with moderate CKD playing a significant role in exacerbating vascular alterations in obese individuals. Further research should explore the molecular mechanisms underlying these observations, and longitudinal studies are also warranted to assess the progression of arterial stiffness, the clinical implications, and the impact of interventions, such as bariatric surgery, on middle-sized arteries remodeling in individuals affected by obesity and kidney impairment.

## ACKNOWLEDGEMENTS

Authors contributions statement: D.M. made substantial contributions to the study's conception, data collection, statistical analysis, and drafting of the manuscript. S.B. contributed to data acquisition. M.N., P.B., and S.T. were involved in critically revising the manuscript for important intellectual content. R.M.B. contributed significantly to the study's design, data collection and interpretation, and manuscript drafting.

Funding: this research did not receive any specific grant from funding agencies in the public, commercial or not-for-profit sectors.

All procedures performed in this study were in accordance with the ethical standards of the institutional and/or national research committee and with the 1964 Helsinki declaration and its later amendments or comparable ethical standards. Informed consent was obtained from all individual participants included in the study.

### Conflicts of interest

There are no conflicts of interest.
